# A Comprehensive Cognitive Health Management Model for Community-Dwelling Older Adults in Shanghai

**DOI:** 10.1093/geroni/igab046.1873

**Published:** 2021-12-17

**Authors:** Jiayuan Qiu, Jing Nie, Yuan Fang, Xia Li

**Affiliations:** 1 Shanghai Mental Health Center, Shanghai, China (People's Republic); 2 Shanghai Jiaotong University School of Medicine, Shanghai, Shanghai, China (People's Republic)

## Abstract

To investigate whether a comprehensive cognitive health management model improves cognitive function among community-dwelling older adults in Shanghai, China. The comprehensive cognitive health management model included brain health screening, individualized brain health consultation and referrals, and annual follow-up monitoring. We compared 161 older adults (43 with MCI, 118 with normal aging) who received the health management model, to an equal size of control group who did not receive the model on their cognition and emotion using Montreal Cognitive Assessment Scale (MoCA) and Geriatric Depression Scale (GDS). Over one-year, participants with MCI in the intervention group showed a significant increase of MoCA score than their MCI counterparts in the control group. Participants with normal aging in the intervention group showed no differences in MoCA and GDS with those in the control group. The individualized cognitive health management model is promising to assist community-dwelling older adults with MCI to maintain brain health.

